# Spatial distribution of tuberculosis and its association with meteorological factors in mainland China

**DOI:** 10.1186/s12879-019-4008-1

**Published:** 2019-05-03

**Authors:** Yingjie Zhang, Mengyang Liu, Samuel S. Wu, Hui Jiang, Junjie Zhang, Songwang Wang, Wei Ma, Qihuan Li, Yuan Ma, Yue Liu, Wei Feng, Endawoke Amsalu, Xia Li, Wei Wang, Weimin Li, Xiuhua Guo

**Affiliations:** 1 Chinese Center for Disease Control and Prevention, Beijing, 102206 China; 2 School of Public Health, Capital Medical University, Beijing, 100069 China; 3 Beijing Municipal Key Laboratory of Clinical Epidemiology, Capital Medical University, Beijing, 100069 China; 4 Department of Statistics, University of Florida, Gainesville, FL 32610-7450 USA; 5 National Tuberculosis Clinical Lab of China, Beijing Chest Hospital, Capital Medical University, Beijing, 101149 China; 6 Beijing Key Laboratory of Drug Resistance Tuberculosis, Beijing Tuberculosis and Thoracic Tumor Research Institute, Beijing, 101149 China; 7 School of life sciences, Beijing Normal University, Beijing, 100875 China; 8 Institute of statistics and big data, Renmin University of China, Beijing, 100872 China; 9 Department of Mathematics and Statistics, La Trobe University, Bundoora, Victoria 3086 Australia; 10 School of Medical Sciences and Health, Edith Cowan University, WA6027, Perth, Australia

**Keywords:** Tuberculosis, Spatial distribution, Meteorological factors, Geographically weighted regression

## Abstract

**Background:**

The incidence of tuberculosis (TB) remains high worldwide. Current strategies will not eradicate TB by 2035; instead, by 2182 is more likely. Therefore, it is urgent that new risk factors be identified.

**Methods:**

An ecological study was conducted in 340 prefectures in China from 2005 to 2015. The spatial distribution of TB incidence was shown by clustering and hotspot analysis. The relationship between the distribution patterns and six meteorological factors was evaluated by the geographically weighted regression (GWR) model.

**Results:**

During the 11 years of the study period, TB incidence was persistently low in the east and high in the west. Local coefficients from the GWR model showed a positive correlation between TB incidence and yearly average rainfall (AR) but a negative correlation with other meteorological factors. Average relative humidity (ARH) was negatively correlated with the incidence of TB in all prefectures (*p* < 0.05).

**Conclusion:**

Meteorological factors may play an important role in the prevention and control of TB.

**Electronic supplementary material:**

The online version of this article (10.1186/s12879-019-4008-1) contains supplementary material, which is available to authorized users.

## Background

Tuberculosis (TB) is still one of the most infectious diseases worldwide with an estimated 10.0 million new cases and 1.3 million deaths according to the Global Tuberculosis Report from 2018 [1]. China is ranked second among the 30 high burden countries for TB and accounted for 9% of the world’s cases. Previous studies indicated that TB epidemics were influenced by three principal aspects: environment, host (human) and pathogen (*M. tuberculosis*) [2, 3]. The environmental aspect mainly refers to the social environment such as socioeconomic level, medical research and demographic factors [4–6]. The prevalence of TB in China has halved since 1991 due to the strategy of short-course chemotherapy following WHO guidelines to control TB [7]. However, if we rely only on the original strategy, the global TB epidemic, including China’s epidemic, will not end by 2035, which is the aim of the World Health Organization (WHO)'s “End TB Strategy”. To complete this aim as early as possible, and to further improve the Chinese TB control strategy, we should pay attention to new or previously neglected risk factors, such as weather [8]. At present, most studies have focused on the relationship between TB incidence and meteorological factors at the provincial level [9–11]. To further control TB incidence, we need to study the association between TB and meteorological factors from a narrower geographical area level. Additionally, biological research suggests that a temperature of 37 °C and sufficient oxygen and water are favoured for the propagation of *M. tuberculosis*, but the pathogen is very sensitive to ultraviolet (UV) light, which is found in sunlight [12]. Therefore, it was speculated that meteorological factors may affect the tuberculosis incidence by affecting the growth and reproduction of *M. tuberculosis* to a certain extent. Therefore, the aim of this study was to explore whether meteorological factors from the prefecture level are potential determinants of TB incidence in China in order to provide a theoretical basis for the prevention and control measures of TB.

## Methods

### Date sources

**Fig. 1:**
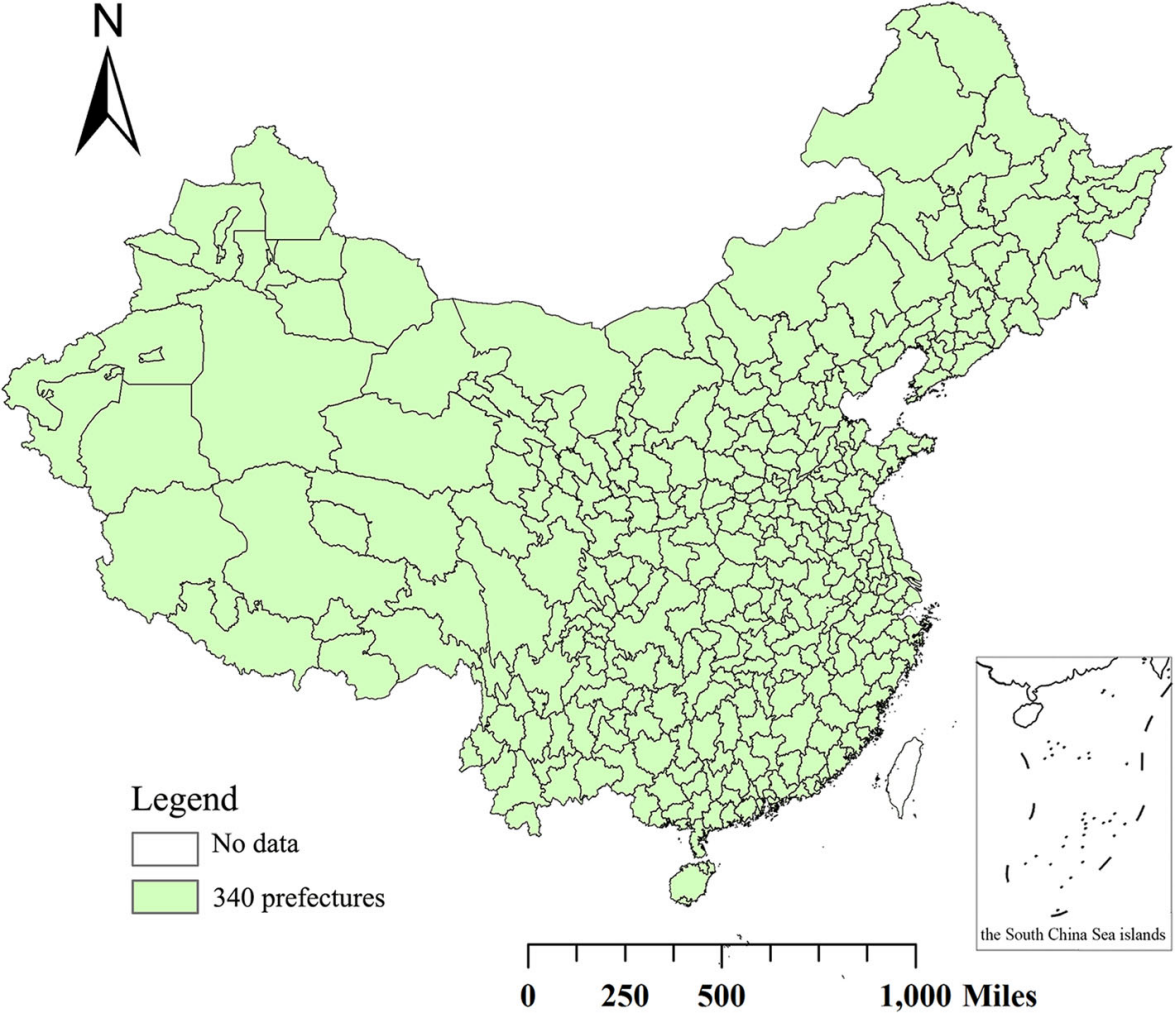
The distribution of 340 prefectures in mainland China

All TB patients were from the Infectious Disease Reporting System (IDRS) and the Tuberculosis Information Management System (TBIMS) from the Chinese Center for Disease Control and Prevention (CCDC) [13, 14], which covers 99.6% of land area of China, including 340 prefectures (Fig. 1). We collected all TB case data from 1 January 2005 to 31 December 2015, including age, sex, address and some clinical diagnostic results.

We also obtained daily meteorological data for each prefecture during the study period from the China Meteorological Data Sharing Service System [15], including the average temperature (AT, °C), average relative humidity (ARH, %), average wind speed (AWS, m/s), average atmospheric pressure (AAP, hPa), average rainfall (AR, mm) and average sunshine duration (ASD, h). In this study, average values from 2005 to 2015 were calculated from the daily values.

Additionally, the size of the population in each prefecture in every year was obtained from the Chinese Statistical Bureau [16], which was used to calculate the TB incidence in each prefecture.

### Statistical analysis

Frequently used descriptive epidemiologic methods were applied to analyse the distribution patterns of TB incidence. The Anselin Local Moran’s I statistic and the Getis-Ord Gi* statistic were used to analyse the clustering of TB incidence and to identify TB incidence hotspots, respectively. The geographically weighted regression (GWR) model was used to explore the spatial variation of the relationship between TB incidence and meteorological factors. Spatial positions of the data were taken into consideration in the GWR model, so the local parameters changed with the spatial position [17]. Variance inflation factors (VIFs) were used to assess multicollinearity among the indicators [18], and we think that a collinearity problem does not exist among the meteorological factors.

A conventional GWR model can be described by the following equation:

$$ {\mathrm{y}}_{\mathrm{j}}={\upbeta}_0\left({\upmu}_{\mathrm{j}},{\upnu}_{\mathrm{j}}\right)+\sum \limits_{\mathrm{i}=1}^{\mathrm{p}}{\upbeta}_{\mathrm{i}}\left({\upmu}_{\mathrm{j}},{\upnu}_{\mathrm{j}}\right){\mathrm{X}}_{\mathrm{i}\mathrm{j}}+{\upvarepsilon}_{\mathrm{j}} $$

where (μ_j_, ν_j_) is the spatial coordinate of sample point j; β_0_(μ_j_, ν_j_) and *β*_*i*_(*μ*_*j*_, *ν*_*j*_) are the regression constant and the regression coefficient of sample point j, respectively; and ε_j_ is the random error of the independent distribution. Considering that the samples were not regularly spaced in 340 prefectures, the adaptive bandwidth and Gaussian kernel were implemented to build the model, which provides the same number of samples for each local estimate and is based on the whitepaper for GWR [19, 20].

The GWR model was built with the GWR4.0 software. All statistical tests were two-sided, and *P*-values less than 0.05 were considered statistically significant.

## Results

A total of 10,271,169 TB cases were reported from 1 January 2005 to 31 December 2015. After excluding cases with missing or abnormal values, our final sample included a total of 10,268,212 TB cases.

### Spatial characteristics of TB incidence

**Fig. 2:**
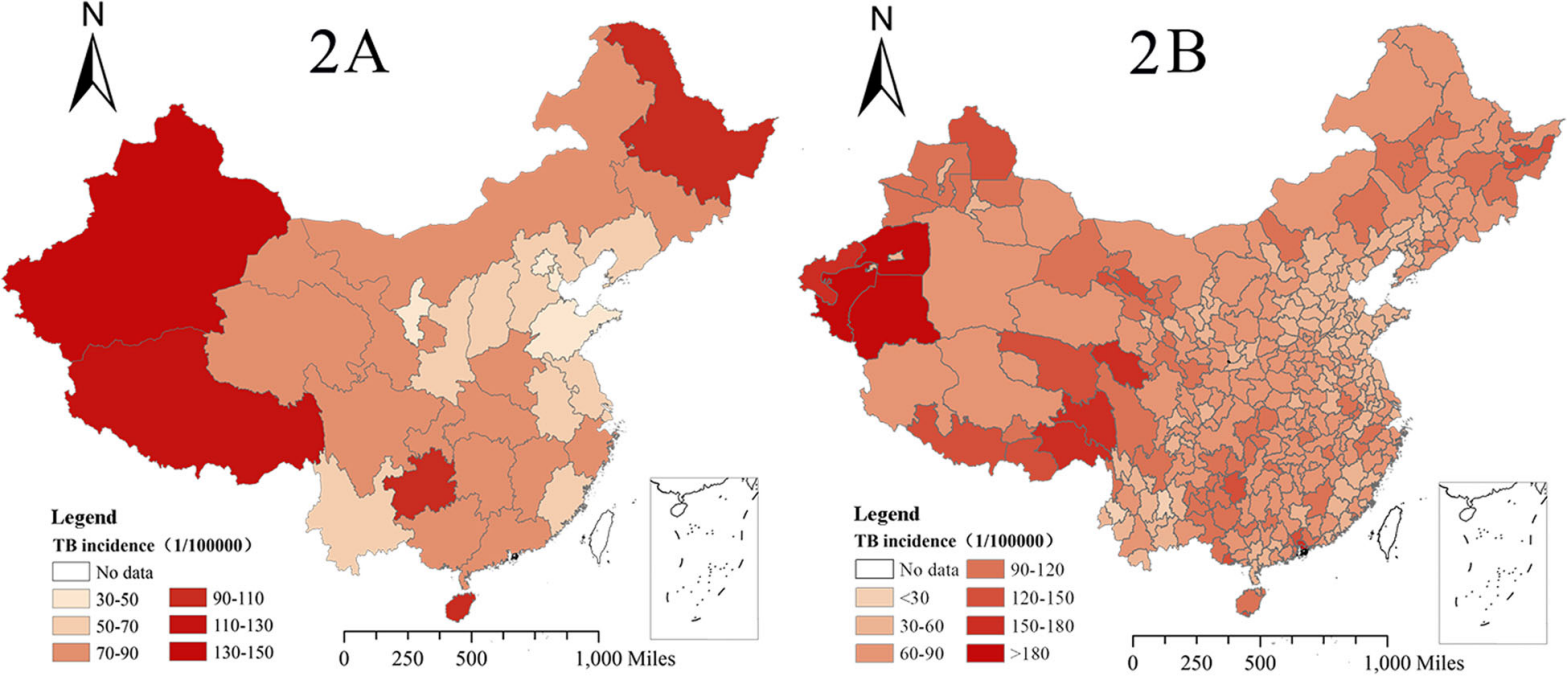
The average annual incidence of TB by province (2a) and by prefecture (2b) in mainland China from 2005 to 2015

In general, the spatial analysis indicated an unbalanced geographical distribution of TB incidence in China, with high rate in the west and a low rate in the east between 2005 and 2015. The Xinjiang Uygur Autonomous Region had the highest incidence among 31 provinces. Specifically, Hotan Prefecture, Kashi Prefecture and Aksu Prefecture, located in the south of the Xinjiang Uygur Autonomous Region, had the highest TB incidence of over 180/100000. Additionally, some other prefectures adjacent to the border between the Tibet Autonomous Region and Gansu Province, Heilongjiang Province, Guizhou Province and Hainan Province also had a high TB incidence (Fig. 2b). In contrast, some areas in eastern China, such as Beijing, Tianjin, Shandong Province and Hebei Province, had relatively low TB incidence (Fig. 2a).

**Fig. 3:**
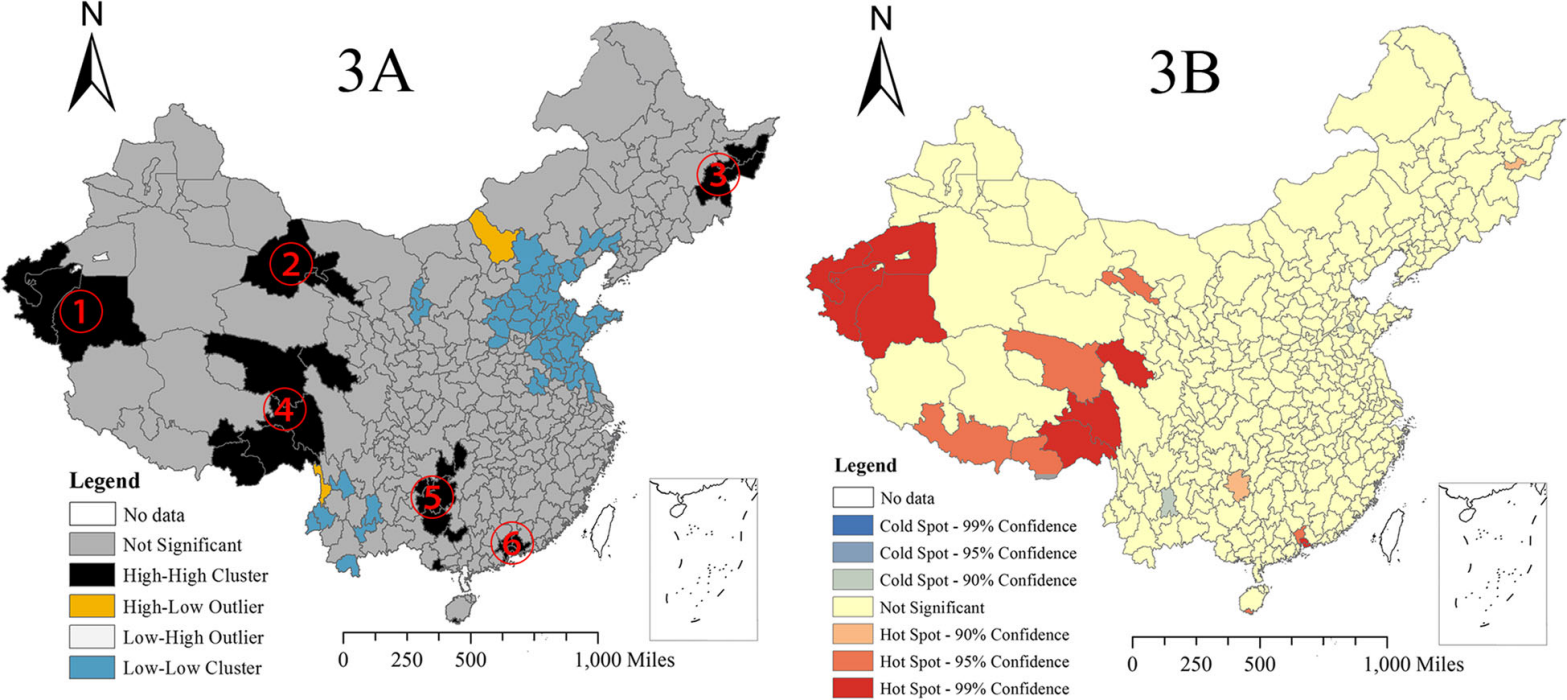
Clusters from the Anselin Local Moran’s I analysis (3a) and hotspots with the Getis-Ord Gi* statistic (3b) for TB incidence from 2005 to 2015

The clustering and hotspot analyses showed the prefectures with relatively high TB incidence, which coincided with the results in the thematic map and helped obtain a more accurate positioning. Six high-high clusters were found by the clustering analysis (Fig. 3a). Area ① was in the south of Xinjiang and included three prefectures. Areas ② and ③ included three prefectures in the north of Gansu and four prefectures in the east of Heilongjiang, respectively. Areas ④ included six prefectures adjacent to the border between Tibet and Qinghai Province, and Area ⑤ included seven prefectures within Hunan, Guizhou and Guangxi. Area ⑥ was in the south of Guangdong Province and consisted of seven prefectures (Fig. 3b).

### Association between TB incidence and meteorological factors

**Fig. 4:**
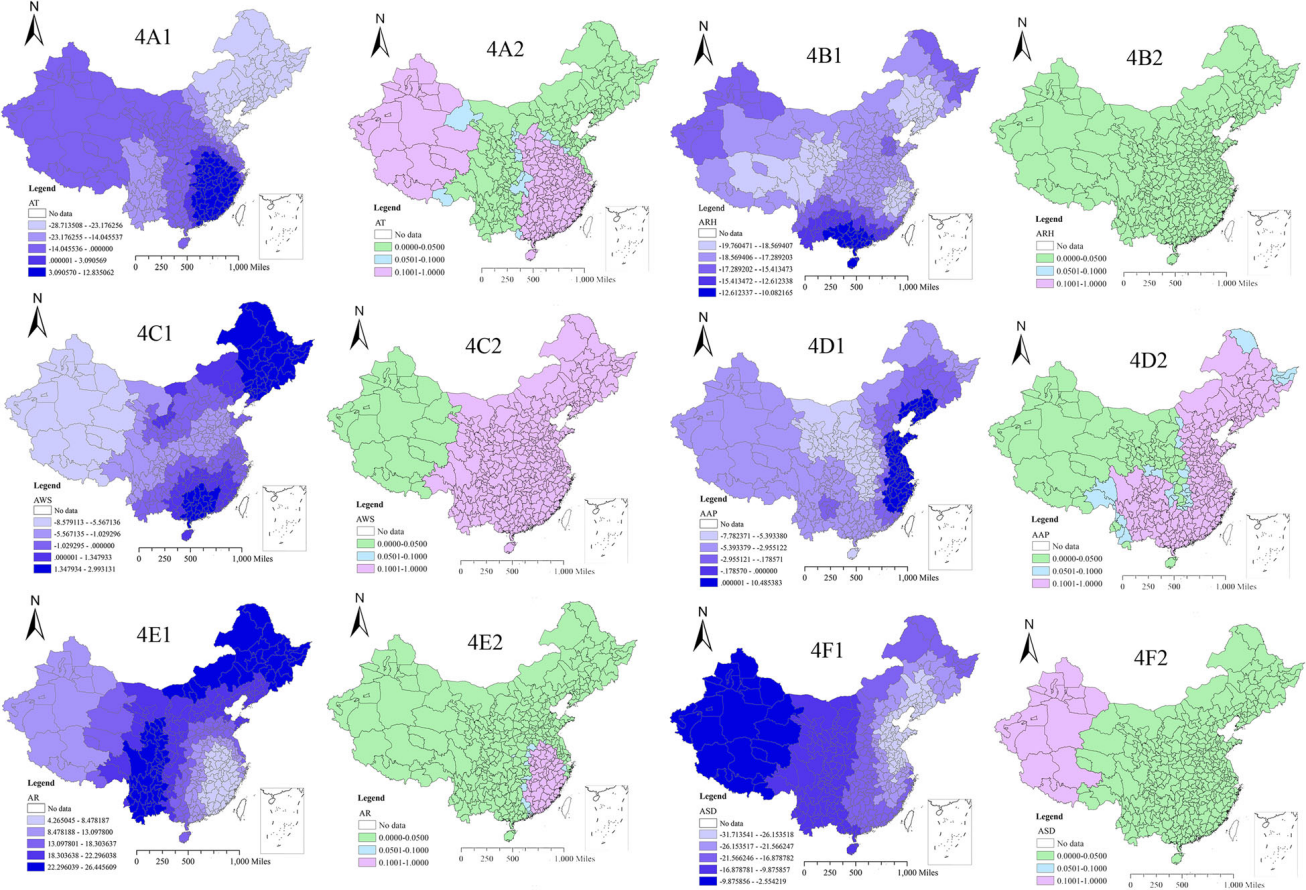
The spatial mapping of local regression coefficients and *p*-values in 340 prefectures based on the GWR model from 2005 to 2015. Figure footnotes: **a** Average temperature (AT, °C) from 2005 to 2015; **b** Average relative humidity (ARH, %) from 2005 to 2015; **c** Average wind speed (AWS, m/s) from 2005 to 2015; **d** Average air pressure (AAP, hPa) from 2005 to 2015; **e** Average rainfall (AR, mm) from 2005 to 2015; **f** Average sunshine duration (ASD, h) from 2005 to 2015. Note: The map Figs. 1, 2, 3 and 4 were depicted by ourselves and were not taken from other source

The GWR model improved the fitness of the regression analysis (adj. R^2^ = 0.3523, AICc = 3146.56) compared with the ordinary least squares regression (OLS) model (adj. R^2^ = 0.1623, AICc = 3227.48). There were large differences in the distribution characteristics of meteorological factors in 340 prefectures (Additional file 1: Figure S1). The GWR model found that the influence effect and influence degree of six meteorological factors on TB incidence had significant spatial variability (Table 1 and Fig. 4). AR (Fig. 4e1) had a positive correlation with TB incidence from 2005 to 2015 while ARH (Fig. 4b1) and ASD (Fig. 4f1) were negatively correlated with TB incidence. The estimated local coefficients of AR, ARH and ASD ranged from 4.27 to 26.45, − 19.76 to − 10.08 and − 31.71 to − 2.55, respectively. Furthermore, ARH was statistically significant in all 340 prefectures (*P* < 0.05), while AR was significant for all prefectures except some in the southeastern China, and ASD was significant for all prefectures except some in the western China. In addition, the relationships between the other three meteorological factors and TB incidence were not consistent across the study area. Specifically, AT was statistically significant only in north-eastern and central China (Fig. 4a1), and AWS and AAP were statistically significant in western China (Fig. 4c1 and Fig. 4d1, respectively), and all of them were negatively correlated.

**Table 1:** The local GWR coefficient between reported TB incidence and meteorological factors in 340 prefectures in 2005–2015

Variable	Min	P_25_	P_50_	P_75_	Max	Range
Intercept	53.05	64.06	67.98	72.05	76.80	23.76
AT^a^	− 28.71	−17.93	−9.82	1.21	12.84	41.55
ARH^a^	−19.76	−18.52	− 17.93	−16.55	− 10.08	9.68
AWS^a^	−8.58	−1.27	−0.51	0.87	2.99	11.57
AAP^a^	−7.78	−5.05	−3.91	−0.75	10.49	18.27
AR^a^	4.27	10.53	16.77	22.03	26.45	22.18
ASD^a^	−31.71	−24.44	−19.12	−14.35	− 2.55	29.16

^a^Table footnotes: ^a^*AT* (centigrade degree) Average temperature from 2005 to 2015, ^a^*ARH* (%) Average relative humidity from 2005 to 2015, ^a^*AWS* (m/s) Average wind speed from 2005 to 2015; ^a^*AAP* (hPa) Average air pressure from 2005 to 2015, ^a^*AR* (mm) Average rainfall from 2005 to 2015, ^a^*ASD* (h) Average sunshine duration from 2005 to 2015

## Discussion

During 2005–2015 in mainland China, the spatial distribution presented a high incidence in the west and a low incidence in the east. The western region with high incidence included the Xinjiang Uygur Autonomous Region, the Tibet Autonomous Region, Heilongjiang Province and Gansu Province. In contrast, the eastern region including Beijing, Tianjin and Shandong Province enjoyed a low TB incidence. The possible reasons for the geographic differences are as follows: first, the western region has a relatively underdeveloped economy, poor medical resources, a low educational level and a high proportion of minorities [21]; in contrast, eastern China has a relatively developed economy, adequate medical resources, a high educational level and a good living and work environment contribute to the low TB incidence. A recent survey of tuberculosis hospitals in China found that 34.6% of TB patients lived in western China, but only 12.8% of hospitals and 14.8% of beds were located in that region in 2010 [4]. In addition to the environment (socioeconomic) and host factors, we can also analyse these factors to observe how they relate to the pathogenic bacteria. *M. tuberculosis* lineage 2 was dominant in the eastern region, while *M. tuberculosis* lineages 2, 3 and 4 were present in the western region [22, 23]. Many studies have suggested that *M. tuberculosis* lineage 2 is more pathogenic than other lineages [22] but the western China presented a more serious condition than the east, which implies that the environment may play a more important role in TB onset than the pathogen itself, so it is meaningful to conduct research on the meteorological factors. Additionally, from the cluster and hotspot analyses, we found that six areas (Fig. 3a) (four areas were in the west (①, ②, ④ and ⑤), one was east of Heilongjiang (③), one was in the Zhujiang Delta, Guangdong (⑥)) had high TB incidence, which is the key areas for TB control and provention.

A correlation analysis with the GWR model showed that AR had a positive correlation with TB incidence. The increased rainfall may create a suitable environment for the growth and reproduction of *M. tuberculosis* [10, 24]. However, a negative correlation was observed between ASD and TB incidence. For ASD, on the one hand, a long sunshine duration with large amounts of UV light would restrict the development of *M. tuberculosis* [25]. On the other hand, the UV light could help the synthesis of vitamin D, which could protect people from TB to a certain extent [26, 27]. Additionally, biological research [12] support our study of meteorological factors across the country; that is, TB incidence is positively correlated with the average precipitation and is negatively corrected with the average sunshine duration.

*M. tuberculosis* is the pathogen of human TB, and humans are hosts. The time from inhalation of *M. tuberculosis* to onset is long for humans, and meteorological factors are not relevant at this stage. However, meteorological factors may play an important role when TB patients expel *M. tuberculosis* into the surrounding environment by spitting. Our study also suggested that ARH is negatively correlated with the incidence of TB in the whole country (*p* < 0.05). In aerosol form only, *M. tuberculosis* can be inhaled into the lung to cause TB. Humanity has reduced dust emissions and the formulation of aerosols, and has thus reduced the spread of TB. In addition, TB incidence was associated with AWS and AAP in many prefectures. The higher AWS could accelerate ventilation, dilute the concentration of bacteria and help reduce the risk of becoming infected. For the AAP, increased atmosphere flow usually forms from high air pressure regions to low air pressure regions, so the mechanism of negative correlation between air pressure and TB incidence may be similar to wind speed, but further explorations are needed.

There are some limitations of the study. First, the potential for under-reporting of cases is inevitable in surveillance data, which is dependent on healthcare-seeking behaviours. In addition, if there is less healthcare available in certain areas, one might expect less reporting, so TB incidence would be underestimated. Second, this is a cross-sectional study with all data collapsed from 2005 to 2015, so the time effects, such as a lag effect, were ignored. Third, we took only meteorological factors into consideration, and some other risk factors associated with TB incidence, such as healthcare access, socioeconomic status and individual-level factors that correlate with geography were not considered due to the unavailability of data. Therefore, further studies should control for other factors affecting TB at a more detailed time scale. Moreover, an ecological study cannot provide conclusive results but can only generate and develop hypotheses.

## Conclusion

In this study, we found that meteorological factors may play an important role in TB incidence in many prefectures in mainland China. Therefore, the prevention and control strategies of TB should take meteorological factors into account. Next, we will proceed to further study about meteorological factors according to the results of this study.

## Additional file

Additional file 1: **Table S1.** The number of reported TB cases in 31 provinces of mainland China, 2005–2015. **Figure S1.** Spatial distribution of six meteorological factors in 340 prefectures from 2005 to 2015. (DOC 1020 kb)

## Abbreviations

AAP: Average atmospheric pressure
AR: Average rainfall
ARH: Average relative humidity
ASD: Average sunshine duration
AT: Average temperature
AWS: Average wind speed
CCDC: Chinese Center for Disease Control and Prevention
GWR: Geographically weighted regression
IDRS: Infectious Disease Reporting System
OLS: Ordinary least squares regression
TB: Tuberculosis
TBIMS: Tuberculosis Information Management System
UV: Ultraviolet
VIF: Variance inflation factor
WHO: World Health Organization
