# Supplementary material for: Spatial distribution of tuberculosis and its association with meteorological factors in mainland China
Source: BMC Infect Dis. 2019 May 3;19:379. doi: 10.1186/s12879-019-4008-1 (PMC6500018; doi:10.1186/s12879-019-4008-1)
Supplement: Supplementary file 1 — Table S1. The number of reported TB cases in 31 provinces of mainland China, 2005–2015. Figure S1. Spatial distribution of six meteorological factors in 340 prefectures from 2005 to 2015. (DOC 1020 kb) [file 12879_2019_4008_MOESM1_ESM.doc]

Supplementary Table 1. The number of reported TB cases in 31 provinces of mainland China, 2005-2015

| Order | Province name | 2005(%) | 2006(%) | 2007(%) | 2008(%) | 2009(%) | 2010(%) | 2011(%) | 2012(%) | 2013(%) | 2014(%) | 2015(%) | Total (%) |
| --- | --- | --- | --- | --- | --- | --- | --- | --- | --- | --- | --- | --- | --- |
| 1 | Beijing | 4078(0.42) | 4446(0.44) | 4630(0.44) | 4873(0.47) | 4781(0.49) | 4802(0.51) | 4497(0.49) | 4289(0.48) | 3841(0.45) | 4076(0.49) | 5732(0.71) | 50045(0.49) |
| 2 | Tianjin | 4304(0.45) | 4332(0.43) | 4523(0.43) | 4532(0.44) | 3419(0.35) | 3563(0.38) | 3632(0.4) | 3234(0.36) | 3557(0.42) | 3254(0.39) | 3492(0.43) | 41842(0.41) |
| 3 | Hebei | 31879(3.31) | 40031(3.95) | 41274(3.97) | 41912(4.06) | 42088(4.28) | 38720(4.13) | 36941(4.05) | 36332(4.04) | 33457(3.91) | 31987(3.87) | 30680(3.82) | 405301(3.95) |
| 4 | Shanxi | 26399(2.74) | 24504(2.42) | 24652(2.37) | 24849(2.41) | 25065(2.55) | 24374(2.6) | 23658(2.59) | 21078(2.34) | 18969(2.22) | 16067(1.94) | 15236(1.89) | 244851(2.38) |
| 5 | Neimenggu | 24321(2.52) | 23979(2.37) | 25945(2.49) | 25080(2.43) | 22321(2.27) | 19488(2.08) | 19027(2.09) | 18242(2.03) | 15392(1.8) | 12007(1.45) | 11148(1.39) | 216950(2.11) |
| 6 | Liaoning | 21033(2.18) | 24521(2.42) | 25765(2.48) | 28145(2.73) | 25628(2.61) | 24659(2.63) | 24794(2.72) | 25379(2.82) | 24555(2.87) | 23263(2.81) | 24825(3.09) | 272567(2.65) |
| 7 | Jilin | 21455(2.22) | 22365(2.21) | 22902(2.2) | 23090(2.24) | 22844(2.32) | 22098(2.36) | 20858(2.29) | 17623(1.96) | 15849(1.85) | 14844(1.8) | 13002(1.62) | 216930(2.11) |
| 8 | Heilongjiang | 35787(3.71) | 39160(3.86) | 41827(4.02) | 41914(4.06) | 37834(3.85) | 35932(3.84) | 33238(3.64) | 32821(3.65) | 33037(3.86) | 33004(3.99) | 33218(4.13) | 397772(3.87) |
| 9 | Shanghai | 6358(0.66) | 7741(0.76) | 8072(0.78) | 8073(0.78) | 7723(0.79) | 7905(0.84) | 8470(0.93) | 8228(0.91) | 8299(0.97) | 8274(1) | 7782(0.97) | 86925(0.85) |
| 10 | Jiangsu | 51001(5.29) | 50166(4.95) | 48944(4.7) | 47639(4.62) | 45008(4.58) | 42891(4.58) | 39245(4.3) | 38792(4.31) | 35293(4.13) | 34373(4.16) | 32253(4.01) | 465605(4.53) |
| 11 | Zhejiang | 39201(4.07) | 42128(4.16) | 42631(4.1) | 40751(3.95) | 38492(3.92) | 36876(3.94) | 36662(4.02) | 35644(3.96) | 32014(3.74) | 32170(3.89) | 30175(3.75) | 406744(3.96) |
| 12 | Anhui | 48754(5.06) | 48653(4.8) | 47440(4.56) | 45439(4.4) | 42280(4.3) | 38052(4.06) | 35285(3.87) | 36219(4.02) | 34595(4.05) | 33961(4.11) | 33108(4.12) | 443786(4.32) |
| 13 | Fujian | 23376(2.42) | 25709(2.54) | 25064(2.41) | 25221(2.44) | 23626(2.4) | 20866(2.23) | 20099(2.2) | 18831(2.09) | 17832(2.09) | 17555(2.12) | 16663(2.07) | 234842(2.29) |
| 14 | Jiangxi | 51588(5.35) | 45634(4.5) | 42728(4.11) | 39902(3.87) | 40041(4.07) | 37940(4.05) | 37345(4.09) | 36243(4.03) | 33557(3.92) | 34117(4.13) | 33832(4.21) | 432927(4.22) |
| 15 | Shandong | 38274(3.97) | 39679(3.91) | 42099(4.05) | 43938(4.26) | 41972(4.27) | 40229(4.29) | 37014(4.06) | 36668(4.07) | 36405(4.26) | 35017(4.24) | 32646(4.06) | 423941(4.13) |
| 16 | Henan | 89102(9.24) | 84273(8.31) | 83432(8.02) | 83420(8.08) | 78473(7.99) | 72412(7.73) | 68428(7.5) | 70447(7.82) | 65035(7.6) | 64238(7.77) | 61409(7.64) | 820669(7.99) |
| 17 | Hubei | 47792(4.96) | 52236(5.15) | 55572(5.34) | 52031(5.04) | 49754(5.06) | 47193(5.04) | 46908(5.14) | 47449(5.27) | 45085(5.27) | 43223(5.23) | 42010(5.22) | 529253(5.15) |
| 18 | Hunan | 52553(5.45) | 53567(5.28) | 54204(5.21) | 54090(5.24) | 55331(5.63) | 55760(5.95) | 56759(6.22) | 58634(6.51) | 58002(6.78) | 56804(6.87) | 55933(6.96) | 611637(5.96) |
| 19 | Guangdong | 60609(6.29) | 67108(6.62) | 70655(6.79) | 70311(6.81) | 73478(7.48) | 74901(8) | 74198(8.14) | 71647(7.96) | 66178(7.74) | 67375(8.15) | 65460(8.14) | 761920(7.42) |
| 20 | Guangxi | 44900(4.66) | 49956(4.93) | 53265(5.12) | 50958(4.94) | 42814(4.36) | 41836(4.47) | 39446(4.33) | 38995(4.33) | 37253(4.36) | 35728(4.32) | 34738(4.32) | 469889(4.58) |

Supplementary Table 1. The number of reported TB cases in 31 provinces of mainland China, 2005-2015

| Order | Province name | 2005(%) | 2006(%) | 2007(%) | 2008(%) | 2009(%) | 2010(%) | 2011(%) | 2012(%) | 2013(%) | 2014(%) | 2015(%) | Total (%) |
| --- | --- | --- | --- | --- | --- | --- | --- | --- | --- | --- | --- | --- | --- |
| 21 | Hainan | 7259(0.75) | 9569(0.94) | 9715(0.93) | 9328(0.9) | 8831(0.9) | 8869(0.95) | 8589(0.94) | 8576(0.95) | 7468(0.87) | 6982(0.84) | 6858(0.85) | 92044(0.9) |
| 22 | Chongqing | 30366(3.15) | 29210(2.88) | 30167(2.9) | 27811(2.7) | 26095(2.66) | 24880(2.66) | 24467(2.68) | 23822(2.65) | 23171(2.71) | 22147(2.68) | 21413(2.66) | 283549(2.76) |
| 23 | Sichuan | 64756(6.72) | 70124(6.92) | 73655(7.08) | 71532(6.93) | 70238(7.15) | 65254(6.97) | 63467(6.96) | 60449(6.71) | 57764(6.75) | 53357(6.46) | 50484(6.28) | 701080(6.83) |
| 24 | Guizhou | 36060(3.74) | 37495(3.7) | 44044(4.23) | 50978(4.94) | 47399(4.82) | 41884(4.47) | 43279(4.75) | 42482(4.72) | 41641(4.87) | 39093(4.73) | 40296(5.01) | 464651(4.53) |
| 25 | Yunnan | 21423(2.22) | 22409(2.21) | 23307(2.24) | 22933(2.22) | 22800(2.32) | 22833(2.44) | 24562(2.69) | 26125(2.9) | 24702(2.89) | 23874(2.89) | 23982(2.98) | 258950(2.52) |
| 26 | Xizang | 2390(0.25) | 2984(0.29) | 3304(0.32) | 4099(0.4) | 4320(0.44) | 3878(0.41) | 4072(0.45) | 4124(0.46) | 4283(0.5) | 4537(0.55) | 4045(0.5) | 42036(0.41) |
| 27 | Shaanxi | 26674(2.77) | 26364(2.6) | 25322(2.43) | 25279(2.45) | 23082(2.35) | 21534(2.3) | 23360(2.56) | 22264(2.47) | 21653(2.53) | 19965(2.42) | 19502(2.43) | 254999(2.48) |
| 28 | Gansu | 17443(1.81) | 24900(2.46) | 25981(2.5) | 25586(2.48) | 23754(2.42) | 22796(2.43) | 22496(2.47) | 19865(2.21) | 18371(2.15) | 17417(2.11) | 14475(1.8) | 233084(2.27) |
| 29 | Qinghai | 4220(0.44) | 4603(0.45) | 4793(0.46) | 4946(0.48) | 4563(0.46) | 4143(0.44) | 3861(0.42) | 3775(0.42) | 3755(0.44) | 2790(0.34) | 3107(0.39) | 44556(0.43) |
| 30 | Ningxia | 2992(0.31) | 3811(0.38) | 3575(0.34) | 3478(0.34) | 3370(0.34) | 3337(0.36) | 2899(0.32) | 2666(0.3) | 2461(0.29) | 2712(0.33) | 2589(0.32) | 33890(0.33) |
| 31 | Xinjiang | 27937(2.87) | 32007(3.16) | 31224(2.98) | 29734(2.89) | 25315(2.57) | 26788(2.86) | 24459(2.69) | 29461(3.25) | 31704(3.71) | 32305(3.92) | 34043(4.23) | 324977(3.17) |
| 32 | the Mainland of China | 964284(100) | 1013664(100) | 1040711(100) | 1031872(100) | 982739(100) | 936693(100) | 912015(100) | 900404(100) | 855178(100) | 826516(100) | 804136(100) | 10268212(100) |

**
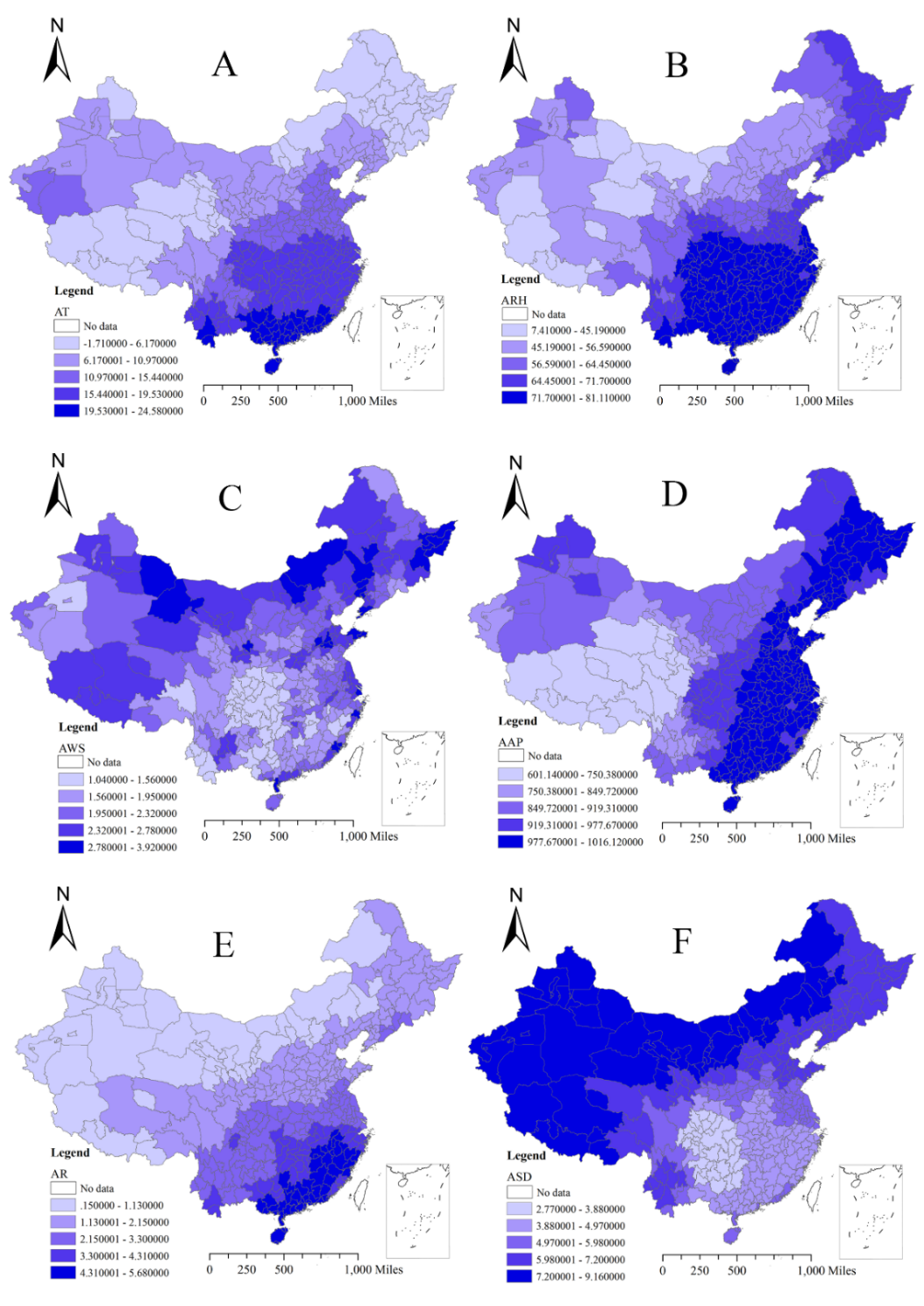
**

Supplementary Figure1. Spatial distribution of six meteorological factors in 340 prefectures from 2005 to 2015

Notes: A. average temperature （AT，centigrade degree）from 2005-2015; B. average relative humidity（ARH，%）from 2005-2015; C. average wind speed（AWS，m/s）from 2005-2015; D. average air pressure （AAP，hPa）from 2005-2015; E. average rainfall （AR，mm）from 2005-2015; F. average sunshine duration（ASD，h）from 2005-2015.
